# *tert*-Butyl *N*-{2-[bis­(prop-2-yn-1-yl)amino]­phen­yl}carbamate

**DOI:** 10.1107/S1600536811016862

**Published:** 2011-05-11

**Authors:** Manavendra K. Singh, Alka Agarwal, Charu Mahawar, Satish K. Awasthi

**Affiliations:** a Department of Medicinal Chemistry, Institute of Medical Sciences, Banaras Hindu University, Varanasi 225 001, U.P. India; b Chemical Biology Laboratory, Department of Chemistry, University of Delhi 110 007, Delhi, India

## Abstract

In the crystal of the title compound, C_17_H_20_N_2_O_2_, the molecules are linked by C—H⋯O interactions. Intra­molecular C—H⋯O and N—H⋯N hydrogen bonds also occur.

## Related literature

For applications of alkyne scaffolds in biology, medicinal and materials chemistry, see: Diederich *et al.* (2005 ▶); Stang & Diederich (1995 ▶); Lam *et al.* (1988 ▶); Patai (1994 ▶). For background to click chemistry, which involves 1,3-dipolar cyclo­addition of an alkyne with an azide and is an efficient and highly versatile tool that has allowed the preparation of a variety of macromolecule conjugates such as sugars, peptides or proteins and DNA, see: Rostovtsev *et al.* (2002 ▶). For the synthesis, see: Lilienkampf *et al.* (2009 ▶). For inter­molecular inter­actions, see: Steiner & Desiraju (1998 ▶). For intra­molecular C—H⋯O hydrogen bonds, see: Smith *et al.* (1993 ▶).
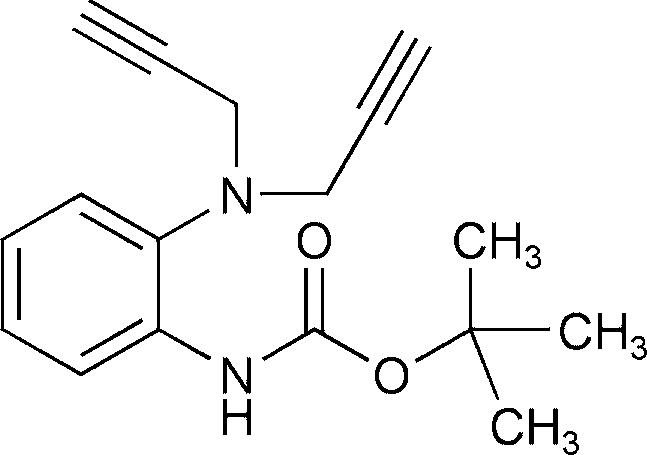


## Experimental

### 

#### Crystal data

C_17_H_20_N_2_O_2_
*M* *_r_* = 284.35
Monoclinic, 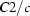

*a* = 19.1936 (12) Å
*b* = 8.7181 (4) Å
*c* = 19.7619 (9) Å
β = 99.513 (5)°
*V* = 3261.3 (3) Å^3^
*Z* = 8
Mo *K*α radiation
μ = 0.08 mm^−1^
*T* = 293 K
0.40 × 0.39 × 0.38 mm

#### Data collection

Oxford Diffraction Xcalibur Eos diffractometer
Absorption correction: multi-scan (*CrysAlis PRO*; Oxford Diffraction, 2009 ▶) *T*_min_ = 0.953, *T*_max_ = 1.000
6969 measured reflections
4389 independent reflections
2427 reflections with *I* > 2σ(*I*)
*R*_int_ = 0.019

#### Refinement

*R*[*F* ^2^ > 2σ(*F* ^2^)] = 0.054
*wR*(*F* ^2^) = 0.156
*S* = 0.96
4389 reflections
194 parameters
H atoms treated by a mixture of independent and constrained refinement
Δρ_max_ = 0.19 e Å^−3^
Δρ_min_ = −0.19 e Å^−3^

### 

Data collection: *CrysAlis PRO* (Oxford Diffraction, 2009 ▶); cell refinement: *CrysAlis PRO*; data reduction: *CrysAlis PRO*; program(s) used to solve structure: *SHELXS97* (Sheldrick, 2008 ▶); program(s) used to refine structure: *SHELXL97* (Sheldrick, 2008 ▶); molecular graphics: *Mercury* (Macrae *et al.*, 2006 ▶); software used to prepare material for publication: *publCIF* (Westrip, 2010 ▶).

## Supplementary Material

Crystal structure: contains datablocks I, global. DOI: 10.1107/S1600536811016862/zj2008sup1.cif

Structure factors: contains datablocks I. DOI: 10.1107/S1600536811016862/zj2008Isup2.hkl

Supplementary material file. DOI: 10.1107/S1600536811016862/zj2008Isup3.cml

Additional supplementary materials:  crystallographic information; 3D view; checkCIF report

## Figures and Tables

**Table 1:** Hydrogen-bond geometry (Å, °)

*D*—H⋯*A*	*D*—H	H⋯*A*	*D*⋯*A*	*D*—H⋯*A*
C10—H10*B*⋯O1^i^	0.97	2.55	3.512 (2)	171
C9—H9⋯O1^ii^	0.93	2.28	3.194 (3)	166
C2—H2⋯O1	0.93	2.32	2.911 (3)	121
N1—H*N*1⋯N2	0.810 (19)	2.28 (2)	2.703 (2)	114 (2)

Symmetry codes: (i) 
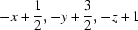
; (ii) 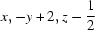.

**Fig. 1.**
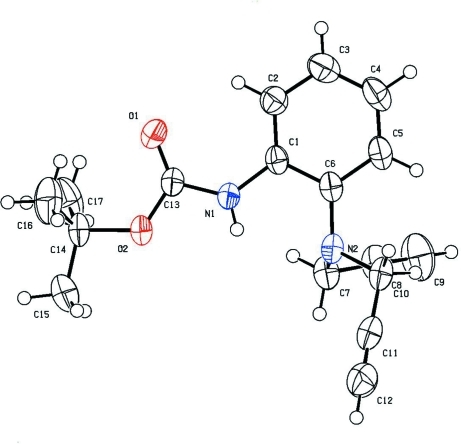
ORTEP diagram of molecule with thermal ellipsoids drawn at 50% probability level Color code: White: C; red: O; blue: N; white: H

**Fig. 2.**
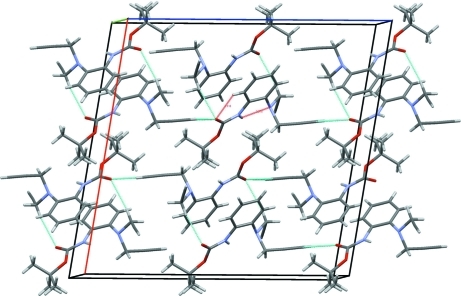
Intermolecular interaction between C—H···O (blue line) and Intramolecular hydrogen bond (red line) showed in packing diagram of molecule along b-plane

**Fig. 3.**
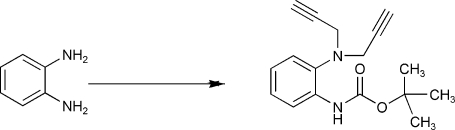
The formation of the title compound.

## supplementary crystallographic information

### Comment

The carbon-carbon triple bond is an important and versatile functional group in organic chemistry. Alkynes are found in numerous natural products as well as in synthetic organic molecules. These alkyne scaffolds have various applications in biology, medicinal and material chemistry (Diederich *et al.*, 2005; Stang & Diederich 1995; Lam *et al.*, 1988; Patai 1994). Click chemistry developed by Sharpless (Rostovtsev *et al.*, 2002) involves 1,3-dipolar cycloaddition of alkyne with azide as an efficient and highly versatile tool that has allowed to prepare a variety of macromolecule conjugates such as sugars, peptides or proteins and DNA. As part of our ongoing work on antimicrobial studies on small molecules, we characterized and report here the crystal structure of [2-(di-prop-2-ynyl-amino)phenyl]carbamic acid *tert*-butyl ester (Figure1).

In the crystal structure, the compound is stabilized by intermolecular interaction between C10—H10B···O1 and C9—H9···O1 (Steiner & Desiraju, 1998) and intramolecular hydrogen bond between C2—H2···O1 (Smith *et al.*, 1993) and N1—HN1···N2 as seen in the crystal packing diagram along *b* axis (Table 1, Figure 2). Considering C1—C6 C13—C15 N1 N2 O1 O2 atom as plane A, C7 C8 C9 atom as plane B, C10 C11 C12 atom as plane C, the dihedral angels between planes A/B, A/C and B/C are 74.74°, 57.52°, 48.94° respectively, suggests that the molecule is not co-planar.

### Experimental

The synthesis of the title compound was carried out according to the published procedure (Lilienkampf, *et al.*, 2009). Briefly, to a solution of (2-aminophenyl)carbamic acid *tert*-butyl ester (1.5 g, 7.2 mmol) in dry acetone was added anhydrous K_2_CO_3_ (7.95 g, 54.6 m mol) and reaction mixture was refluxed for 15–30 minutes. Subsequently, KI (0.60 g m, 3.6 mmol) and propargyl bromide (0.75 ml, 7.8 mmol) were added and further refluxed the reaction mixture for 18 hrs. The reaction mixture was cooled, filtered, and the filtrate was evaporated *in vacuo* to give the product. The crude product was purified by column chromatography using hexane and dichloromethane (65:35) as eluent. The purified product was recrystallized from hexane-dichloromethane (1:1). The colourless crystals were obtained by slow evaporation of solvent at room temperature in several days. Yield: 20%.

^1^H NMR (CDCl_3_): 8.11 (bs, 1H, NH), 7.56–7.55 (m, 1H, Ar—H), 7.35- 7.32 (m, 1H, Ar—H), 7.19–7.14 (m, 1H, Ar—H), 6.99–6.94 (m, 1H, Ar—H), 3.83 (s, 4H, CH_2_), 2.28 (s, 2H, CH), 1.51 (s, 9H, 3xCH_3_).

### Refinement

All H atoms were located from difference Fourier map (range of C—H = 0.93 - 0.97 Å, and N–H = 0.81 Å) and allowed to refine freely.

### Crystal data

**Table:**

C_17_H_20_N_2_O_2_	*F*(000) = 1216.0
*M**_r_* = 284.35	*D*_x_ = 1.158 Mg m^−^^3^
Monoclinic, *C*2/*c*	Mo *K*α radiation, λ = 0.71073 Å
Hall symbol: -C 2yc	Cell parameters from 4389 reflections
*a* = 19.1936 (12) Å	θ = 3.2–29.0°
*b* = 8.7181 (4) Å	µ = 0.08 mm^−^^1^
*c* = 19.7619 (9) Å	*T* = 293 K
β = 99.513 (5)°	Block, colourless
*V* = 3261.3 (3) Å^3^	0.40 × 0.39 × 0.38 mm
*Z* = 8	

### Data collection

**Table:**

Oxford Diffraction Xcalibur Eos diffractometer	4389 independent reflections
Radiation source: fine-focus sealed tube	2427 reflections with *I* > 2σ(*I*)
graphite	*R*_int_ = 0.019
ω scans	θ_max_ = 29.1°, θ_min_ = 3.2°
Absorption correction: multi-scan (*CrysAlis PRO*; Oxford Diffraction, 2009)	*h* = −24→7
*T*_min_ = 0.953, *T*_max_ = 1.000	*k* = −10→10
6969 measured reflections	*l* = −23→26

### Refinement

**Table:**

Refinement on *F*^2^	Primary atom site location: structure-invariant direct methods
Least-squares matrix: full	Secondary atom site location: difference Fourier map
*R*[*F*^2^ > 2σ(*F*^2^)] = 0.054	Hydrogen site location: inferred from neighbouring sites
*wR*(*F*^2^) = 0.156	H atoms treated by a mixture of independent and constrained refinement
*S* = 0.96	*w* = 1/[σ^2^(*F*_o_^2^) + (0.0754*P*)^2^ + 1.325*P*] where *P* = (*F*_o_^2^ + 2*F*_c_^2^)/3
4389 reflections	(Δ/σ)_max_ = 0.05
194 parameters	Δρ_max_ = 0.19 e Å^−^^3^
0 restraints	Δρ_min_ = −0.19 e Å^−^^3^

### Special details

**Table:**

Geometry. All e.s.d.'s (except the e.s.d. in the dihedral angle between two l.s. planes) are estimated using the full covariance matrix. The cell e.s.d.'s are taken into account individually in the estimation of e.s.d.'s in distances, angles and torsion angles; correlations between e.s.d.'s in cell parameters are only used when they are defined by crystal symmetry. An approximate (isotropic) treatment of cell e.s.d.'s is used for estimating e.s.d.'s involving l.s. planes.
Refinement. Refinement of *F*^2^ against ALL reflections. The weighted *R*-factor *wR* and goodness of fit *S* are based on *F*^2^, conventional *R*-factors *R* are based on *F*, with *F* set to zero for negative *F*^2^. The threshold expression of *F*^2^ > σ(*F*^2^) is used only for calculating *R*-factors(gt) *etc*. and is not relevant to the choice of reflections for refinement. *R*-factors based on *F*^2^ are statistically about twice as large as those based on *F*, and *R*- factors based on ALL data will be even larger.

### Fractional atomic coordinates and isotropic or equivalent isotropic displacement parameters (Å^2^)

**Table:**

	*x*	*y*	*z*	*U*_iso_*/*U*_eq_	
HN1	0.1292 (11)	0.706 (2)	0.4601 (9)	0.038 (5)*	
N1	0.13858 (9)	0.78587 (19)	0.47962 (8)	0.0397 (4)	
O2	0.05794 (7)	0.71231 (15)	0.53892 (6)	0.0505 (4)	
N2	0.17096 (8)	0.68525 (17)	0.35922 (7)	0.0399 (4)	
C1	0.18983 (9)	0.8707 (2)	0.45262 (8)	0.0362 (4)	
O1	0.11848 (8)	0.93110 (16)	0.57014 (6)	0.0548 (4)	
C13	0.10630 (10)	0.8202 (2)	0.53354 (8)	0.0380 (4)	
C6	0.20658 (10)	0.8197 (2)	0.38966 (8)	0.0383 (4)	
C2	0.22421 (11)	0.9975 (2)	0.48421 (10)	0.0454 (5)	
H2	0.2130	1.0324	0.5256	0.055*	
C11	0.19018 (12)	0.4288 (3)	0.31791 (9)	0.0510 (5)	
C5	0.25792 (11)	0.8980 (2)	0.36113 (10)	0.0507 (5)	
H5	0.2691	0.8655	0.3194	0.061*	
C10	0.21577 (11)	0.5866 (2)	0.32379 (9)	0.0476 (5)	
H10A	0.2168	0.6274	0.2783	0.057*	
H10B	0.2637	0.5879	0.3489	0.057*	
C8	0.10589 (12)	0.7963 (3)	0.25187 (10)	0.0586 (6)	
C3	0.27519 (12)	1.0729 (2)	0.45480 (11)	0.0544 (5)	
H3	0.2980	1.1581	0.4765	0.065*	
C7	0.10173 (11)	0.7167 (2)	0.31671 (9)	0.0491 (5)	
H7A	0.0771	0.6202	0.3064	0.059*	
H7B	0.0739	0.7786	0.3431	0.059*	
C14	0.00843 (11)	0.7249 (2)	0.58831 (9)	0.0484 (5)	
C4	0.29225 (12)	1.0225 (2)	0.39370 (12)	0.0576 (6)	
H4	0.3270	1.0727	0.3744	0.069*	
C12	0.17318 (14)	0.2999 (3)	0.31334 (12)	0.0681 (7)	
H12	0.1597	0.1974	0.3097	0.082*	
C15	−0.03735 (16)	0.5849 (3)	0.57144 (14)	0.0852 (9)	
H15A	−0.0629	0.5935	0.5255	0.128*	
H15B	−0.0701	0.5772	0.6031	0.128*	
H15C	−0.0081	0.4949	0.5750	0.128*	
C9	0.11141 (16)	0.8551 (4)	0.20009 (13)	0.0838 (8)	
H9	0.1158	0.9020	0.1587	0.101*	
C16	0.04878 (15)	0.7145 (4)	0.66033 (11)	0.0833 (9)	
H16A	0.0750	0.6203	0.6656	0.125*	
H16B	0.0163	0.7169	0.6924	0.125*	
H16C	0.0808	0.7996	0.6689	0.125*	
C17	−0.03353 (15)	0.8709 (3)	0.57635 (15)	0.0854 (9)	
H17A	−0.0585	0.8727	0.5300	0.128*	
H17B	−0.0021	0.9572	0.5837	0.128*	
H17C	−0.0668	0.8759	0.6076	0.128*	

### Atomic displacement parameters (Å^2^)

**Table:**

	*U*^11^	*U*^22^	*U*^33^	*U*^12^	*U*^13^	*U*^23^
N1	0.0456 (10)	0.0393 (9)	0.0381 (8)	−0.0030 (7)	0.0187 (7)	−0.0042 (7)
O2	0.0517 (9)	0.0599 (8)	0.0465 (7)	−0.0109 (7)	0.0271 (6)	−0.0086 (6)
N2	0.0396 (9)	0.0496 (9)	0.0328 (7)	0.0035 (7)	0.0129 (6)	0.0014 (6)
C1	0.0340 (10)	0.0386 (9)	0.0379 (9)	0.0065 (8)	0.0118 (7)	0.0075 (7)
O1	0.0565 (9)	0.0634 (9)	0.0486 (7)	−0.0082 (7)	0.0212 (6)	−0.0186 (7)
C13	0.0358 (10)	0.0462 (10)	0.0332 (8)	0.0030 (8)	0.0094 (7)	0.0006 (7)
C6	0.0362 (10)	0.0424 (10)	0.0386 (9)	0.0062 (8)	0.0126 (7)	0.0083 (7)
C2	0.0431 (11)	0.0422 (10)	0.0530 (11)	0.0036 (9)	0.0133 (8)	−0.0008 (8)
C11	0.0530 (13)	0.0616 (14)	0.0412 (10)	0.0101 (11)	0.0160 (9)	−0.0043 (9)
C5	0.0506 (13)	0.0553 (12)	0.0518 (11)	0.0017 (10)	0.0250 (9)	0.0092 (9)
C10	0.0457 (12)	0.0610 (13)	0.0391 (9)	0.0088 (10)	0.0155 (8)	−0.0007 (8)
C8	0.0522 (14)	0.0731 (15)	0.0499 (12)	0.0075 (12)	0.0066 (9)	0.0173 (10)
C3	0.0455 (13)	0.0434 (11)	0.0756 (14)	−0.0007 (10)	0.0141 (10)	0.0011 (10)
C7	0.0418 (12)	0.0638 (13)	0.0432 (10)	0.0022 (10)	0.0113 (8)	0.0076 (9)
C14	0.0456 (12)	0.0621 (12)	0.0435 (10)	0.0018 (10)	0.0247 (8)	0.0024 (9)
C4	0.0466 (13)	0.0529 (12)	0.0794 (14)	−0.0020 (10)	0.0287 (11)	0.0122 (11)
C12	0.0762 (18)	0.0616 (15)	0.0727 (15)	0.0037 (13)	0.0307 (13)	−0.0087 (11)
C15	0.081 (2)	0.095 (2)	0.0936 (18)	−0.0271 (16)	0.0533 (15)	−0.0110 (15)
C9	0.081 (2)	0.108 (2)	0.0634 (15)	0.0147 (17)	0.0150 (13)	0.0378 (15)
C16	0.086 (2)	0.121 (2)	0.0472 (13)	0.0107 (18)	0.0253 (12)	0.0175 (13)
C17	0.0630 (17)	0.093 (2)	0.110 (2)	0.0268 (15)	0.0427 (15)	0.0300 (16)

### Geometric parameters (Å, °)

**Table:**

N1—C13	1.352 (2)	C8—C7	1.471 (3)
N1—C1	1.404 (2)	C3—C4	1.374 (3)
N1—HN1	0.801 (19)	C3—H3	0.9300
O2—C13	1.338 (2)	C7—H7A	0.9700
O2—C14	1.475 (2)	C7—H7B	0.9700
N2—C6	1.438 (2)	C14—C17	1.504 (3)
N2—C7	1.476 (2)	C14—C16	1.507 (3)
N2—C10	1.472 (2)	C14—C15	1.509 (3)
C1—C2	1.382 (3)	C4—H4	0.9300
C1—C6	1.407 (2)	C12—H12	0.9300
O1—C13	1.207 (2)	C15—H15A	0.9600
C6—C5	1.392 (3)	C15—H15B	0.9600
C2—C3	1.384 (3)	C15—H15C	0.9600
C2—H2	0.9300	C9—H9	0.9300
C11—C12	1.170 (3)	C16—H16A	0.9600
C11—C10	1.459 (3)	C16—H16B	0.9600
C5—C4	1.374 (3)	C16—H16C	0.9600
C5—H5	0.9300	C17—H17A	0.9600
C10—H10A	0.9700	C17—H17B	0.9600
C10—H10B	0.9700	C17—H17C	0.9600
C8—C9	1.164 (3)		
			
C13—N1—C1	128.60 (16)	C8—C7—H7A	108.7
C13—N1—HN1	118.4 (14)	N2—C7—H7A	108.7
C1—N1—HN1	113.0 (14)	C8—C7—H7B	108.7
C13—O2—C14	122.13 (14)	N2—C7—H7B	108.7
C6—N2—C7	114.07 (15)	H7A—C7—H7B	107.6
C6—N2—C10	113.60 (15)	O2—C14—C17	110.24 (16)
C7—N2—C10	112.31 (14)	O2—C14—C16	109.50 (18)
C2—C1—C6	119.35 (16)	C17—C14—C16	112.2 (2)
C2—C1—N1	124.15 (16)	O2—C14—C15	102.07 (16)
C6—C1—N1	116.50 (16)	C17—C14—C15	111.9 (2)
O1—C13—O2	125.71 (16)	C16—C14—C15	110.5 (2)
O1—C13—N1	125.54 (17)	C5—C4—C3	119.92 (19)
O2—C13—N1	108.74 (15)	C5—C4—H4	120.0
C5—C6—C1	118.91 (18)	C3—C4—H4	120.0
C5—C6—N2	123.34 (16)	C11—C12—H12	180.0
C1—C6—N2	117.72 (15)	C14—C15—H15A	109.5
C3—C2—C1	120.53 (18)	C14—C15—H15B	109.5
C3—C2—H2	119.7	H15A—C15—H15B	109.5
C1—C2—H2	119.7	C14—C15—H15C	109.5
C12—C11—C10	176.5 (2)	H15A—C15—H15C	109.5
C4—C5—C6	120.96 (19)	H15B—C15—H15C	109.5
C4—C5—H5	119.5	C8—C9—H9	180.0
C6—C5—H5	119.5	C14—C16—H16A	109.5
C11—C10—N2	111.90 (16)	C14—C16—H16B	109.5
C11—C10—H10A	109.2	H16A—C16—H16B	109.5
N2—C10—H10A	109.2	C14—C16—H16C	109.5
C11—C10—H10B	109.2	H16A—C16—H16C	109.5
N2—C10—H10B	109.2	H16B—C16—H16C	109.5
H10A—C10—H10B	107.9	C14—C17—H17A	109.5
C9—C8—C7	177.1 (3)	C14—C17—H17B	109.5
C4—C3—C2	120.3 (2)	H17A—C17—H17B	109.5
C4—C3—H3	119.8	C14—C17—H17C	109.5
C2—C3—H3	119.8	H17A—C17—H17C	109.5
C8—C7—N2	114.21 (17)	H17B—C17—H17C	109.5
			
C13—N1—C1—C2	10.1 (3)	N1—C1—C2—C3	178.67 (18)
C13—N1—C1—C6	−170.50 (17)	C1—C6—C5—C4	0.4 (3)
C14—O2—C13—O1	5.5 (3)	N2—C6—C5—C4	−177.51 (18)
C14—O2—C13—N1	−173.54 (16)	C12—C11—C10—N2	−149 (4)
C1—N1—C13—O1	−2.8 (3)	C6—N2—C10—C11	157.11 (15)
C1—N1—C13—O2	176.26 (16)	C7—N2—C10—C11	−71.6 (2)
C2—C1—C6—C5	0.5 (3)	C1—C2—C3—C4	0.0 (3)
N1—C1—C6—C5	−178.92 (16)	C9—C8—C7—N2	46 (6)
C2—C1—C6—N2	178.52 (16)	C6—N2—C7—C8	70.5 (2)
N1—C1—C6—N2	−0.9 (2)	C10—N2—C7—C8	−60.6 (2)
C7—N2—C6—C5	−96.4 (2)	C13—O2—C14—C17	57.2 (3)
C10—N2—C6—C5	34.0 (2)	C13—O2—C14—C16	−66.8 (2)
C7—N2—C6—C1	85.68 (18)	C13—O2—C14—C15	176.18 (18)
C10—N2—C6—C1	−143.86 (16)	C6—C5—C4—C3	−1.1 (3)
C6—C1—C2—C3	−0.7 (3)	C2—C3—C4—C5	0.9 (3)

### Hydrogen-bond geometry (Å, °)

**Table:**

*D*—H···*A*	*D*—H	H···*A*	*D*···*A*	*D*—H···*A*
C10—H10B···O1^i^	0.97	2.55	3.512 (2)	171
C9—H9···O1^ii^	0.93	2.28	3.194 (3)	166
C2—H2···O1	0.93	2.32	2.911 (3)	121
N1—HN1···N2	0.810 (19)	2.28 (2)	2.703 (2)	114 (2)

Symmetry codes: (i) −*x*+1/2, −*y*+3/2, −*z*+1; (ii) *x*, −*y*+2, *z*−1/2.
